# The Shenzhen Declaration on Plant Sciences: Too late or just in time?

**DOI:** 10.3897/phytokeys.86.20712

**Published:** 2017-09-18

**Authors:** W. John Kress, Sandra Knapp

**Affiliations:** 1 Editor-in-Chief of PhytoKeys, Distinguished Scientist and Curator of Botany, National Museum of Natural History, Smithsonian Institution, P.O. Box 37012, Washington, DC 20013-7012, USA; 2 Deputy Editor-in-Chief of PhytoKeys, Head of Algae, Fungi and Plants Division, Department of Life Sciences, Natural History Museum, Cromwell Road, London SW7 5BD, United Kingdom

On 29 July 2017 in the closing session of the XIX International Botanical Congress held in Shenzhen, China, nearly 7,000 plant scientists from 77 countries unanimously endorsed a statement to focus their research and educational efforts on finding solutions to the growing problems of “environmental degradation, unsustainable resource use, and biodiversity loss.” This moment was the culmination of a seven-day conference that brought together botanists from around the world to discuss and share their latest exciting research findings on a wide range of topics across the plant sciences. Yet, in addition to the scientific advances being communicated, an awareness and recognition was pervasive and much discussed throughout the week of the IBC that our planet is changing in substantial ways that will affect the social, political, and economic frameworks of our lives into the foreseeable future. And all agreed that these immense changes are the result of unbridled human activities.

The Shenzhen Declaration, conceived and composed by a broadly representative group of scientists, aims to raise the awareness of botanists to the accelerating rate of environmental change around the globe. More importantly it calls on them to make a commitment to take action now in both their lifestyles and their research programs to find solutions before an environmental threshold is crossed that will inevitably lead to irreversible degradation of our societies, natural habitats, and biodiversity. Although many scientists believe that humanity and the planet may have already crossed that threshold, the authors of the Declaration and the botanists who have endorsed it believe that time still exists for answers to be found and implemented. However, that time is short.

The seven priorities outlined in the Declaration range broadly from increased action by scientific communities, more cooperation and integration across disciplines, the implementation of new technologies, valuing local and traditional knowledge, to greater engagement with the public. Realizing and achieving these priorities will be a major challenge that will require re-orienting research agendas and new resources. However, the enthusiastic response to the Declaration by the thousands of plant scientists in Shenzhen suggests that the botanical community now has a solid and inspiring roadmap for the future. If we are to successfully build a green and sustainable Earth, all scientists and citizens, not just botanists, should carefully read, study, and take steps to participate in collective action to make the seven priorities of the Shenzhen Declaration a reality for our common future.

